# Polyamino acid calcified nanohybrids induce immunogenic cell death for augmented chemotherapy and chemo-photodynamic synergistic therapy

**DOI:** 10.7150/thno.64354

**Published:** 2021-09-21

**Authors:** Wei Qiu, Mengyun Liang, Yuan Gao, Xuelian Yang, Xingyao Zhang, Xiaoli Zhang, Peng Xue, Yuejun Kang, Zhigang Xu

**Affiliations:** 1 Key Laboratory of Luminescence Analysis and Molecular Sensing (Southwest University), Ministry of Education, School of Materials and Energy and Chongqing Engineering Research Center for Micro-Nano Biomedical Materials and Devices, Southwest University, Chongqing 400715, P. R. China.; 2 Pediatric Research Institute, Department of Hematology and Oncology, Shenzhen Children's Hospital, Shenzhen, Guangdong 518038, P. R. China.; 3 Key Laboratory of Laser Technology and Optoelectronic Functional Materials of Hainan Province, College of Chemistry and Chemical Engineering, Hainan Normal University, Haikou 571158, P. R. China.

**Keywords:** Calcified nanohybrids, multimodal nanotheranostics, chemotherapy, chemo-photodynamic therapy, immunogenic cell death

## Abstract

**Background:** Monotherapy for cancer treatment is limited by unstable efficacy and uncontrollable toxic side effects, while the multifunctional nanoplatform with complex preparation process cannot avoid the potential toxicity of each functional component.

**Methods:** We exploited tumor-specific activated polyamino acid calcified nanoparticles (CHC NPs) as new-type oxidative stress amplification of anticancer drugs *via* building a safe and biodegradable multifunctional nanoplatform. Giving priority to chemotherapy, and synergizing chemodynamic therapy (CDT) with photodynamic therapy (PDT), this strategy was to achieve enhanced chemotherapy, simultaneously inducing immunogenic cell death and inhibiting tumor cell invasion.

**Results:** Based on amorphous calcium carbonate, pH-responsive nanocarrier was prepared with classical chemotherapeutic drug 10-hydroxycamplothecin (HCPT) and photosensitizer Chlorin e6 (Ce6) to realize multifunctional nanotheranostics.

**Conclusion:** Inventive calcified nanohybrids, where topoisomerase inhibited by HCPT to prevent DNA synthesis, the generation of •OH induced *via* Fenton reaction, along with a large amount of ^1^O_2_ produced by Ce6, might be a promising strategy for anti-tumor combination therapy in clinical translation.

## Introduction

Cancer has always examined the resilience of human beings. As cancer burden increases with years, malignancy continues to be one of the major threats to human life and health 1-3. Aiming to seek accurate diagnosis and effective treatment remains the primary mission in nanomedicine researches, and the development of multimodal nanoplatforms to satisfy the demands of personalized treatment and significant efficacy 4, 5. Constructed by assembling multiple components with unique advantages, multifunctional nanotheranostic platform is still subject to the complicated preparation process and poor repeatability 6. Outmoded nanomedicine exhibits bad biodegradability and is prone to trigger long-term toxicity for patients, which impedes the transformation into clinical practice 7. Exploitation for degradable and multifunctional nanomedicine with prominent biocompatibility and simple synthesis process has become an urgent need to achieve breakthroughs in cancer therapy. Calcium carbonate (CaCO_3_), as a typical mineral widely existing in organisms and nature, is expected to participate in the construction of multimodal nanoplatforms due to its biodegradation performance, excellent biocompatibility, and low cost, as well as great potentials for drug delivery, protein transportation and gene delivery 8-11. The pH sensitivity of CaCO_3_ makes it one of the attractive drug candidates for responsive release in the tumor microenvironment to achieve controlled release without drug leakage 12, 13. According to frontier researches, crystal calcium carbonates with micron size have trapped into some inherent limitations, which may hinder its application as a drug delivery system, even affect transfer efficiency 14. Amorphous calcium carbonate breaks this impasse, and high solubility enables it to be better absorbed from the blood into cells, thus having greater absorbability and higher bioavailability 15, 16. Additionally, amorphous calcium carbonate also possesses multiple functions such as microenvironmental pH regulated, local anti-inflammatory activity enhanced, immunity improved, and cancer cell metabolism influenced. Previous reports manifested that amorphous calcium carbonate-based nanodrug delivery platform could directionally release the cargo at the tumor site due to sensitive pH response 17. Calcified nanosubstrate like this can be easy to carry various types of bioactive molecules without a fussy and complicated formation process, which indicates that it's potential to combine with diverse modifications or organic ligands. Introduction of PEGylation agent, like PEG-P(Glu) block copolymer is anticipated to further ameliorate biological function and biocompatibility of composite nanodrugs 18.

In addition to the development of various treatment methods and biocompatible nanomaterials, the improvement of tumor treatment effect is also inseparable from the discovery and application of clinical treatment mechanisms 19, 20. At present, the therapeutic target of most clinical tumor administration is apoptosis-related pathway. 10-hydroxycamplothecin (HCPT), extracted from the unique plant of Camptotheca acuminata in China, is a classic and effective anti-tumor chemotherapy drug, and has been widely used in clinical practice in many countries. Compared with camptothecin, HCPT has better curative effect and less side effects in the treatment of malignancy. HCPT is also a semi-synthetic precursor compound of camptothecin anticancer drugs CPT-11 and TPT, permitted by FDA for clinical use. HCPT is a kind of natural alkaloid with broad spectrum, high efficiency and low toxicity, which participates in anti-tumor by inhibiting topoisomerase I (TOPO I) activity to interfere with the DNA replication of tumor cells 21, 22. As a promising alternative therapy, chemodynamic therapy (CDT) produces reactive oxygen species (ROS) in specific tumor microenvironment, which is followed by Fenton reaction (2Fe^3+^ + H_2_O_2_ → 2Fe^2+^ + O_2_ + 2H^+^, Fe^2+^+ H_2_O_2_ → Fe^3+^ + (OH)^-^ + •OH), catalyzing the conversion of low-toxic H_2_O_2_ to highly toxic hydroxyl radical (•OH), and significantly elevating the ROS level in tumor cells 23-25. Based on the reductive tumor microenvironment, the introduced ferric ions would be translated into ferrous ions to participate in Fenton reaction. Fenton reaction could play a better role under acidic condition, and ROS generated in TME is easily consumed by the high level of intracellular glutathione (GSH) 26. Recently, phototherapy of tumor cells damaged by phototoxicity has attracted great attention in the field of tumor therapy owing to the presence of advantages such as less invasion, less adverse effects, strong temporal and spatial selectivity 27-29. Photodynamic therapy (PDT) applies photosensitizers (PSs) for transforming laser into singlet oxygen ^1^O_2_ as substrate to injure tumor cells. As a commonly applied photosensitizer for clinical imaging and diagnose, Chlorin e6 (Ce6) is suitable for the exploitation of photodynamic therapy upon tumors thanks to high efficiency in producing ^1^O_2_ 30, 31. However, like most photosensitizers so far, Ce6 is hydrophobic and tends to aggregate in aqueous solution, which has some difficulties in practical application. Properly speaking, the efficacy of single phototherapy is far away from our satisfactory goal in cancer nanomedicine 32-34. Moreover, ^1^O_2_ produced by most photosensitizers (PSs) are heavily oxygen-dependent in PDT, and rapid depletion of molecular oxygen can further aggravate hypoxia 35. Therefore, tumor hypoxia has become a major obstacle to PDT treatment. Clinical treatment-activated immunogenic cell death (ICD) can reinforce tumor immunotherapy by stimulating the autoimmune system through secreting associated signals. At present, therapeutic means like chemotherapy and phototherapy can induce ICD, with cytotoxic •OH and ^1^O_2_ involved in regulation ICD 36-38. In consequence, increased intracellular ROS levels are highly proposed to amplify ICD and PDT could effectively arouse immunogenicity during treatment 39, 40.

**Scheme 1:**
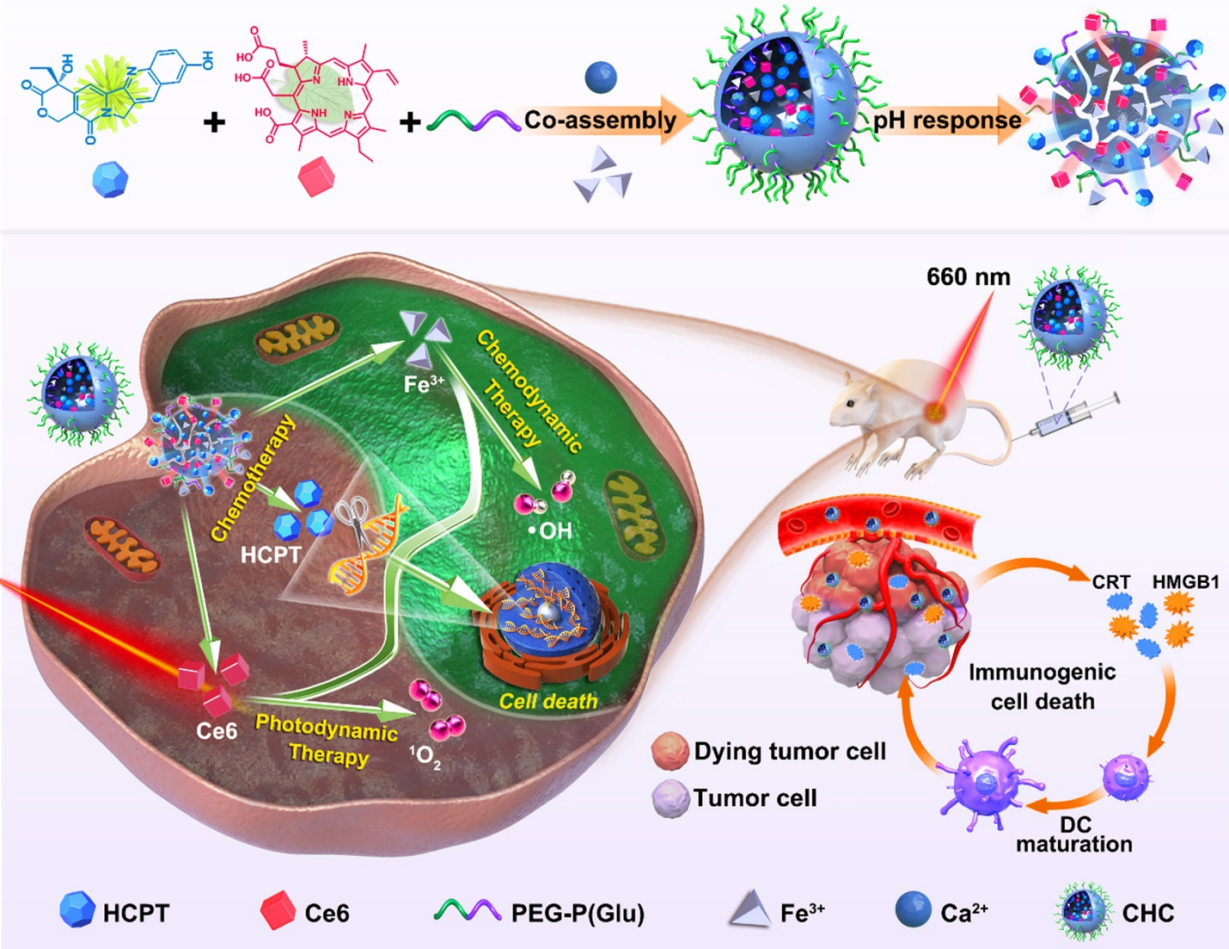
Schematic diagram about fabrication of CHC NPs and anticancer mechanism of multimodal therapies based on chemotherapy and CDT/PDT therapy.

Although chemotherapy is the main method of cancer treatment, both CDT based on ROS involvement in cell homeostasis and local treatment PDT as non-invasive therapy have also been widely investigated 41, 42. With high level of H_2_O_2_ in cancer cells, CDT is specific to TME and thus minimizes adverse reaction on healthy tissue, compared with conventional PDT 43. On the basis of the preliminary studies, chemotherapy agent (HCPT) and photosensitizer (Ce6) were incorporated into calcified nanohybrids (denoted as CHC NPs) to amplify oxidative stress. CHC NPs were fabricated through the complexation between drugs and amorphous calcium carbonate nanosubstrate, as well as tailored modification with PEG-P(Glu). The improved and optimized nanoparticle stability and drug release property, were likely to determine the efficacy of nanodrug in the body. As illustrated in Scheme 1, CDT utilized the Fenton reaction to produce •OH, while PDT produced ^1^O_2_ under laser irradiation to trigger tumor-associated apoptosis or necrosis. ROS derived from PDT caused mitochondrial dysfunction and accelerated the end of tumor cells during the process of immunogenic cell death (ICD). The efforts made by CDT and PDT were aimed at enhancing HCPT-based chemotherapy, making up for monotherapy shortcomings, and realizing the construction of multimode nanoplatform.

## Experimental

### Materials

10-Hydroxy camptothecin (HCPT, 98%) was purchased from Adamas-beta (China). Chlorin e6 (Ce6, 92%) was supplied by Yuanye Biotechnology Ltd (China). Calcium chloride anhydrous (CaCl_2_), iron (III) chloride hexahydrate (FeCl_3_∙6H_2_O), sodium carbonate (Na_2_CO_3_), tris-hydrochloride buffer and HEPES were acquired from Aladdin (China). Antibodies for chromatin-binding protein high mobility group B1 (HMGB1) and Calreticulin were provided by cell signaling technology (China). All other agents were supplied by Life Technologies (Carlsbad, USA) and used directly.

### Preparation and characterization of CHC NPs

The formation of CHC nanohybrids referred to typical methods as previously reported with some modification 44. Briefly, 1 mM Tris-HCl buffer (pH 7.6) containing 100 mM CaCl_2_ and 20 mM FeCl_3_ was dropwise added to 50 mM HEPES buffer (pH 7.1) according to the optimal solvent ratio of 1:2. The HEPES buffer encompassed 10 mM Na_2_CO_3_, HCPT and Ce6 (each 400 μg), as well as 5 mg PEG-P(Glu) constructed as formerly described 18, 45. Further, the obtained mixture was continuously stirred at 25 °C for 24 h and centrifugated respectively at 3000 rpm and 13000 rpm for 5 min to remove excess ions, drugs and copolymers.

### Cumulative drug release from CHC NPs

Due to the weak acidity in tumor microenvironment (TME), the release behavior of CHC NPs under different pH condition *in vitro* was studied by dialysis to simulate the real TME* in vivo*. Typically, 1.0 mL CHC was set in a 3.5 kDa dialysis bag and put into 15 mL buffer (pH 5.0, pH 6.5, pH 7.4, respectively). At different timepoints, 1 mL original buffer was collected and replaced by corresponding fresh buffer of consistent volume. Eventually, the concentration of Ce6 and HCPT released were determined by microplate reader (Tecan SPARK-10M).

### Biosafety of CHC NPs

#### L929 culture

Biocompatibility of CHC NPs was initially evaluated by murine L929 fibroblasts (L929 cells). After adherence, cells were cultured with the medium including CHC NPs at Ce6 concentration of 1~100 µg/mL. Subsequent to incubation for 24 h, 100 µL MTT solution was contained into per well. The supernatant was discarded after 4 h reaction and filled with 150 µL DMSO. Then, optical absorption intensity was observed *via* microplate reader.

#### Hemolysis analysis

After whole blood was centrifuged at 3000 rpm, erythrocytes (RBC) were harvested and subsequently rinsed with PBS for three times. Afterwards, 0.3 mL of CHC NPs at different Ce6 concentration was introduced into the tube containing the diluent RBC for incubation for 2 h at 37 °C. Finally, the absorbance of supernatant was measured at 570 nm.



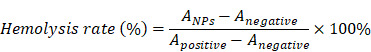



#### Blood routine test

6-7 weeks Balb/c mice were intravenously injected with PBS buffer, HCPT, Ce6 or CHC NPs dispersion (100 µL, 1 mg/mL). Next, blood specimen was collected from the eye socket at day 1, 7 and 14. Key blood indexes were measured by automatic hematology analyzer (NC-2600Vet, Mindary, China).

### Cellular uptake and colocalization analysis

Initially, flow cytometry was applied to measure the phagocytosis of CT26 towards CHC NPs. In short, CT26 cells (7×10^4^/well) were respectively exposed to medium containing CHC NPs (Ce6, 5 μg/mL) for 0.5 h, 1 h, 2 h, 4 h and 6 h. For colocalization assessment, after treated with CHC NPs (Ce6, 10 μg/mL) at various time points, CT26 cells were stained with Lyso-Tracker and Mito-Tracker reagents on behalf of lysosomes and mitochondria separately, finally recorded by confocal laser scanning microscope (CLSM 880, Carl Zeiss, Germany).

### Cytotoxicity assessment *in vitro*

First of all, cytotoxicity of CHC NPs to CT26 cells was evaluated on the basis of standard MTT assay. CT26 cells in 96 well plates (1×10^4^ per well) were cultured overnight prior to the addition of HCPT, Ce6 or CHC NPs for 24 h. Additionally, living/dead cell staining upon FDA/PI was carried out to direct observation of cell survival status and then quantified through Image J software. To analyze cell migration after different administration, CT26 cells in 12-well plate were incubated with HCPT, Ce6 and CHC NPs for 24 h. Subsequently, the cells (5×10^4^ per well) treated as described above were transferred to upper chambers to visualize cell migration. Metastatic cells were viewed and imaged with the fluorescence microscope and calculated using Image J software. Cell migration rate (%) = N_treatment_ / N_PBS_ × 100%. (All laser tests were exposed to 660 nm irradiation after cultivation with drugs for 4 h).

### ROS generation capacity

To monitor ^1^O_2_ generation *in vitro*, DPBF (1 mg/mL) was mixed with CHC NPs (Ce6: 5 μg/mL), irradiated by 660 nm laser every minute (180 mW/cm^2^). The UV absorption change of DPBF at 400 nm was determined at the corresponding time point above. MB (1 mg/mL) probe was applied to detect the production of •OH under various condition. Afterwards, intracellular ROS generation performance of various treatment towards CT26 was conducted. Specially, the cells were stained by DCFH-DA for 30 min with or without 660 nm laser irradiation for 3 min, while the nuclei were processed by Hoechst 33342. Furthermore, the generation of ^1^O_2_ was measured by SOSG, which could specifically react with ^1^O_2_ and produce green fluorescence that were observed upon CLSM. Besides, electron spin resonance (ESR) was utilized to validate the generation of ^1^O_2_ and •OH by CHC NPs in presence of H_2_O_2_, to illustrate the type of ROS generation in detail.

### Immunofluorescence imaging of CHC NPs in DNA damage

Attributed to HCPT combination with topoisomerase to suppress the synthesis of DNA, this assay was exploited to evaluate the DNA damage of CHC NPs to tumor cells based on measuring the intensity of green fluorescence. Adherent CT26 cells were separately exposed to HCPT, Ce6 and CHC NPs at 37 °C for 4 h. After replacing with fresh medium, they were subjected to 660 nm laser irradiation or not and cultured for an additional 8 h. Through formaldehyde fixed for 20 min, Triton X-100 permeabilized for 20 min and BSA blocked for 2 h, the cells were incubated with the primary antibody γ-H2AX at 4 °C overnight for DNA damage detection. Replaced with the Alexa Fluor 488-secondary antibody, cells were finally visualized by CLSM. In addition, tumor sections in each group after treatment were used to further immunofluorescence detection to verify DNA damage *in vivo*.

### Immunologic cell death (ICD) induced by CHC NPs

According to the performance of HMGB1 and CRT on the surface of tumor cells, immunofluorescence (IF) analysis was employed to estimate ICD induced by CHC NPs *in vitro*. Concretely, CT26 cells 5×10^4^ were added into 12-well plates overnight and respectively treated by HCPT, Ce6 and CHC NPs for 24 h, with 660 nm irradiation or not. After rinsed with PBS, fixed, permeated and blocked in turns, then cultured with primary antibodies of anti-HMGB1 and anti-CRT at 4 °C overnight, cells were replaced with AF488-labeled secondary antibodies for 1 h and stained by DAPI for 10 min, finally tested by CLSM. In another aspect, solid tumors of each group were sliced into sections for HMGB1 and CRT immunofluorescence detection. By way of deparaffinized, dehydrated, heat induced epitope retrieval with citrate buffer (pH = 6.0) and closure by 10% goat serum in 37 °C, tumor sections were seperately incubated with primary and secondary antibodies, as same as the processs of intracellular detection of CRT and HMGB1* in vitro*. All immunofluorescence staining slices were scanned by CLSM.

### Penetration capacity of CHC NPs in CT26 MCSs

The manifestation of drug release behavior and vertical tumor penetration ability to CHC NPs were assessed by using CT26 multicellular spheroids (MCSs). On basis of previous reports 46, 1×10^3^ CT26 cells were planted into 96-well plates containing 50 µL hot agarose and incubated for 3-5 days to constitute MCSs. After replaced by fresh medium including CHC NPs (Ce6, 15 µg/mL), CT26 MCSs were cultured for 2 h, 6 h and 12 h respectively. Lastly, the photographs of different depth were captured by CLSM.

### NIR imaging and biodistribution

Balb/c mice inoculated with 4T1 cells were randomly divided into CHC group and Ce6 group (n = 3) to evaluate the biodistribution of CHC NPs. Once tumor volume arrived approximate 200 mm^3^, CHC NPs and free Ce6 at the equivalent Ce6 dosage were intravenously injected into mice. The mice NIR images were recorded by IVIS Lumina imaging system at different timepoints and the distribution of Ce6 in the tumor and organs were observed after 48 h.

### Tumor suppression efficiency *in vivo*

CT26 cells were subcutaneously inoculated on 6-7 weeks Balb/c mice to establish CT26 xenograft models. Each animal process was executed obeying the administrative provisions of Southwest University ethics committee. Until tumor volume reached ~200 mm^3^, all mice were separated into 5 treatment groups at random (PBS, HCPT, CHC NPs, Ce6+L and CHC NPs+L in a single dose of 5.0 mg/kg of HCPT or 3 mg/kg of Ce6). Tumors were exposed to 660 nm laser (180 mW/cm^2^) after 24 h administration. After 3 times administration and NIR laser irradiation, the mice were sacrificed and tumor tissue sections and major organs were taken for H&E staining and further analyzed for Ki67 and TUNEL assay. Tumor volume was reckoned in accordance with the classic formula: length×width^2^ / 2.

### Statistical analysis

All results are displayed as the mean ± standard deviation (SD). Statistical significance (*p < 0.05, **p < 0.01, ***p < 0.001) was performed by Student's t-test for two-group comparisons. Image J software was applied for various quantification.

## Results and Discussion

### Formation and characterization of CHC nanohybrids

**Figure 1:**
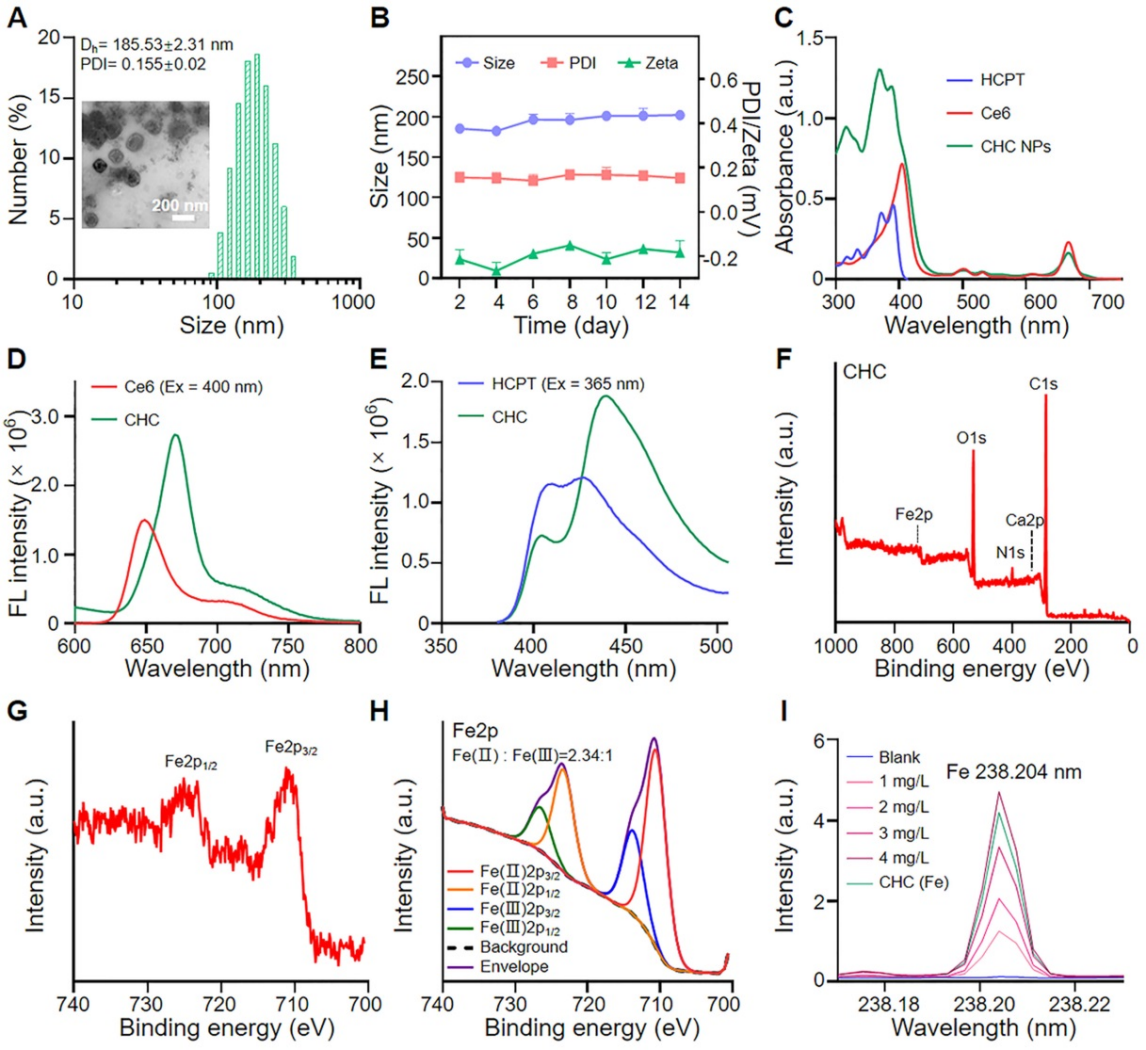
Physiochemical characterization of CHC NPs. (A) Hydrodynamic diameter and TEM image of CHC NPs. (B) Size, zeta potentials and polydispersity of CHC NPs dispersed in PBS during 2 weeks. (C) UV-vis absorption of Ce6, HCPT and CHC NPs. Fluorescence spectra of (D) Ce6 and (E) HCPT in CHC NPs. (F) XPS full survey spectrum of CHC NPs. (G) Core level XPS spectrum of Fe2p and (H) high-resolution Fe2p spectrum. (I) Quantitative analysis of Fe element in CHC NPs using ICP-MS.

Built upon various unique material capabilities, the carboxyl terminus of PEG-P(Glu) complexed with Ca^2+^ in the aqueous phase served as the core, and hydrophilic PEG chains formed the outside of CHC NPs upon hydrophilic and hydrophobic interactions, so that both hydrophobic chemotherapeutics HCPT and photosensitizer Ce6 were loaded inside of calcium carbonate nanoparticles (denoted CHC NPs) with stirring. Synergizing multiple modes of cancer therapy was successful, including HCPT-based chemotherapy to inhibit DNA replication, Fenton reaction for CDT and the mass production of ^1^O_2_ from PDT. HCPT and Ce6 following the optimal ratio were loaded into CHC NPs. The loading efficiency of HCPT in CHC nanohybrids was 8.6%, while to Ce6 was 5.1%, respectively, implying the high drug-loading capability of CHC NPs. The size of CHC NPs was 163.33 ± 15.45 nm or 185.53 ± 2.31 nm (polydispersity index, PDI = 0.155 ± 0.02) with approximately spherical morphology, respectively measured by transmission electron microscopy (TEM) and dynamic light scattering (DLS) (Figure 1A). The diameter and PDI of CHC nanoparticles were suitable so as to possess the excellent stability in PBS (pH 7.4) with or without 10% FBS for long-term storage over 2 weeks (Figure 1B and S1), and a small amount negative charge distributed on the surface of CHC might prolong circulation in the blood (Figure S2). Due to pH sensitivity of CaCO_3_, the diameter of CHC NPs in acid PBS (pH 5.0) was recorded by DLS in Figure S3, which further manifested that CHC was pH-responsive nanoparticle, promising to release drugs in response to acidic tumor microenvironment. The presence of HCPT and Ce6 embedded in CHC was confirmed by UV-vis shown in Figure 1C. As exhibited in Figure 1D-E and Figure S4, fluorescence spectra and FT-IR spectra further proved the successful preparation of CHC.

The chemical valence of Ca and Fe elements were monitored by X-ray photoelectron spectroscopy (XPS) 47, 48, which showed the Ca(II) peak at 346.68 eV was exactly corresponding to the binding energy of Ca2p (Figure S5), Fe(III) at 713.68 eV and 726.48 eV along with Fe(II) at 710.53 eV and 723.29 eV were indexed to the binding energy of Fe2p (Figure 1F-H). As depicted in Figure 1I, the existence of Fe element in CHC NPs was further determined by inductively coupled plasma mass spectrometry (ICP-MS). To explore the decomposition of CHC NPs at different pH values, the release patterns of Ce6 and HCPT from CHC were simulated in weak acidic environment *via* dialysis. As described in Figure S6, CHC NPs revealed sustained slow release properties. More Ce6 and HCPT were dissociated from CHC NPs at the environment of pH = 5.0 or pH = 6.5, while only a few were separated from the normal condition of pH = 7.4, which suggested that CHC NPs were sensitive response to acidic tumor microenvironment, but no response to the normal.

### CHC NPs as an oxidative stress amplifier

**Figure 2:**
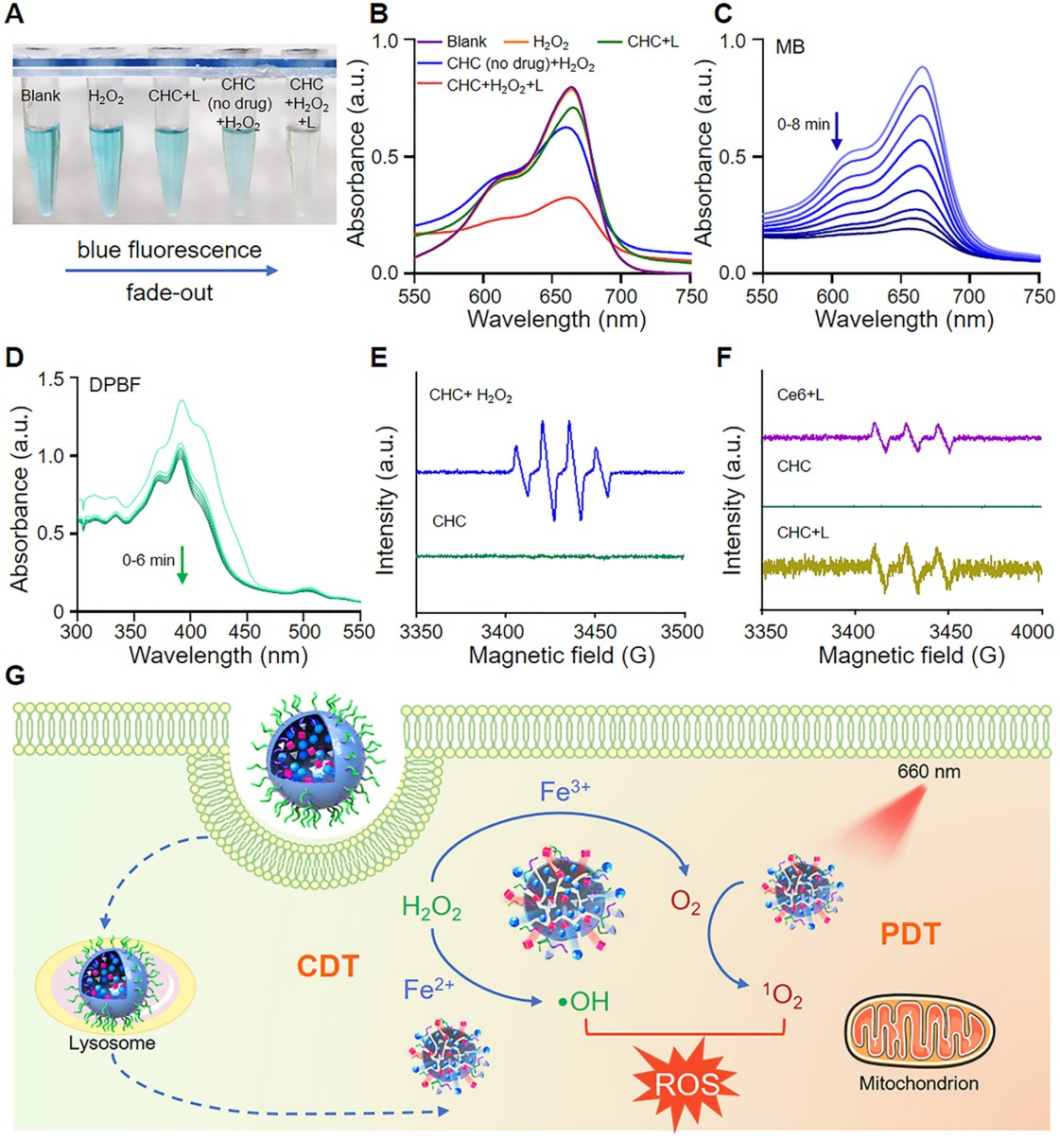
Detection of ROS generation from CHC NPs. (A) Visual color variation of aqueous samples including MB. (B) •OH generation detected by MB probe upon different conditions. (C) UV-vis spectra of MB after reacting with CHC NPs upon 660 nm irradiation for various periods. (D) ^1^O_2_ emergence caught by DPBF probe subject to 660 nm irradiation for up to 6 min. ESR spectra of (E) DMPO/•OH and (F) TEMP/^1^O_2_ adducts in different solutions. (G) Illustration of drug release and robust radical generation.

Methylene blue (MB) was employed to verify the generation of •OH, where both CHC NPs with or without drug could activate Fenton reaction to make MB oxidized and color changes occur (Figure 2A-B). The absorbance of MB at 665 nm reduced with irradiation time which was observed by ultraviolet spectrophotometer (Figure 2C). To prove ^1^O_2_ generation capacity of CHC NPs outside tumor cells upon 660 nm irradiation, the change of DPBF probe was recorded by UV-vis to confirm the large amount ROS yield (Figure 2D). Electron spin resonance (ESR) was employed to verify the existence of ^1^O_2_ or •OH instantaneously caught by 2,2,6,6-tetramethylpiperidine (TEMP) and 5,5-dimethyl-1-pyrroline N-oxide (DMPO), respectively. As demonstrated in Figure 2E and S7, the peak of •OH with intensity ratio of 1:2:1 was produced by CHC NPs *in vitro* with the addition of H_2_O_2_, while CHC+L group exhibited obvious characteristic peak with intensity ratio of 1:1:1 corresponding to the standard peak of 2,2,6,6-Tetramethyl-1-piperidinyloxy (TEMPO) in Figure 2F. The above results indicated that CHC NPs could significantly enhance oxidative stress by igniting the produce of •OH and ^1^O_2_ (Figure 2G).

### Cytotoxicity of CHC NPs *in vitro*

**Figure 3:**
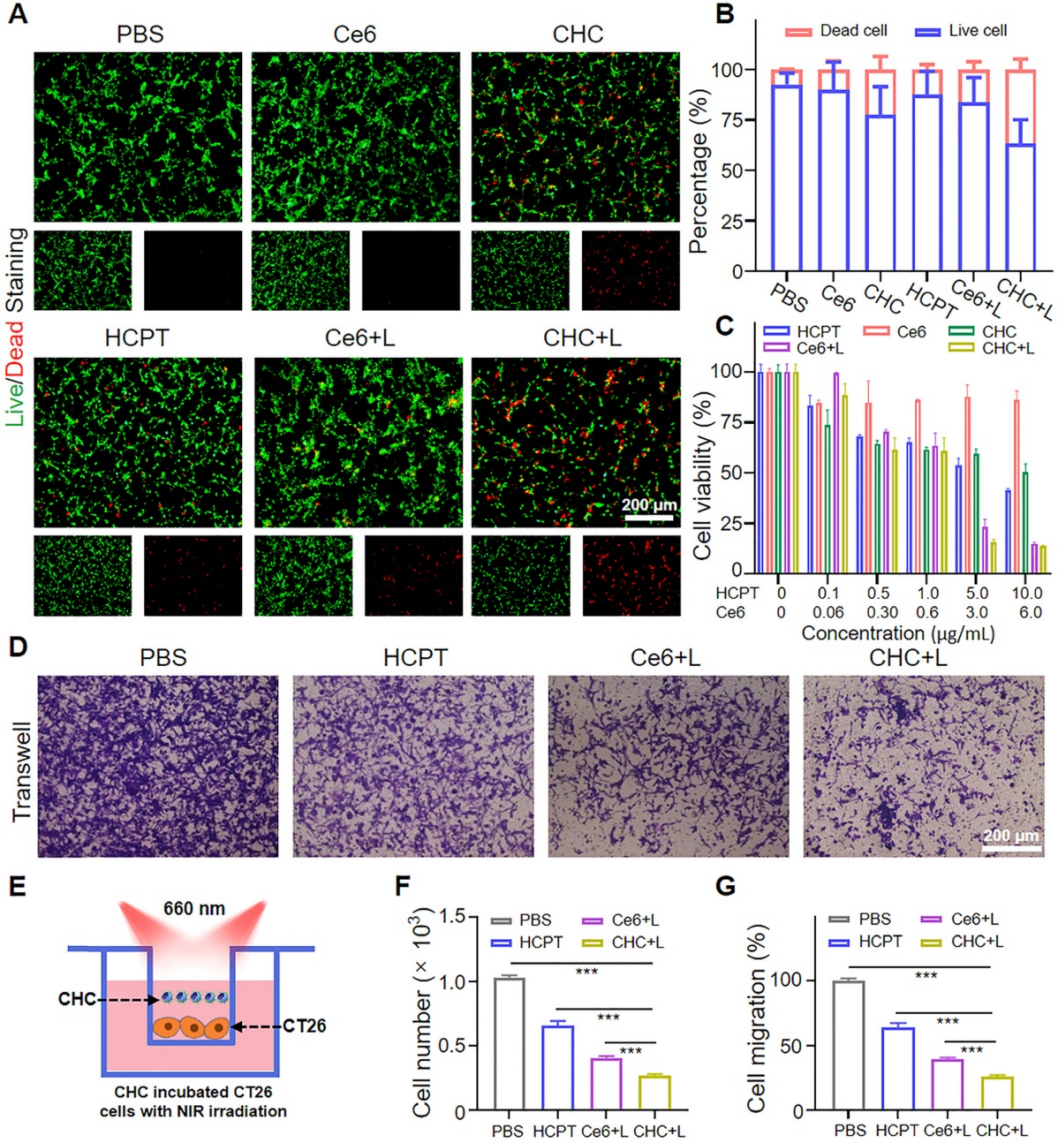
Cytotoxicity evaluation of CHC NPs. (A) FDA (green)/PI (red) staining images of CT26 cells under various treatment for 12 h (Ce6 concentration: 10 µg/mL) and (B) quantification of living or dead cells area. (C) Viability of CT26 cells incubated in various groups. (D) CT26 cells migration influenced by PBS, HCPT, Ce6+L and CHC+L. (E) Illustration of transwell system experiment. The calculation of (F) cell number and (G) cell migration rate. Data denoted as mean ± SD, n = 3.

FDA/PI staining was employed to observed the green living cells or red dead cells *via* fluorescence imaging to evaluate the cytotoxicity of various drugs visually (Figure 3A-B). Apart from that, MTT assay was applied to further manifest that CHC NPs represented the excellent cytotoxicity to CT26 tumor cells, where the cell viability was less than 15% treated with CHC+L (660 nm, 180 mW/cm^2^) at Ce6 of 6 μg/mL (Figure 3C). By virtue of Chou-Talalay method 49, the combination index (CI) was calculated to 0.635, which indicated that the co-delivery of HCPT and Ce6 enhanced the inhibition effifacy (CI < 1: synergistic effect; CI = 1: additive effect, CI > 1: antagonism effect). Due to synergized HCPT and Ce6, the consistance phenomenon emerged in Transwell assay (Figure 3D-E). As exhibited in Figure 3F and 3G, the migration capacities of CT26 cells were influenced by the treatment of PBS, HCPT, Ce6+L or CHC+L. Especially, CHC+L significantly inhibited the movement of CT26 cells from the upper to the lower chamber, corresponding migration rate as low as 26%.

### Oxidative stress amplification-enhanced CDT and PDT therapy fuel DNA damage-based chemotherapy

**Figure 4:**
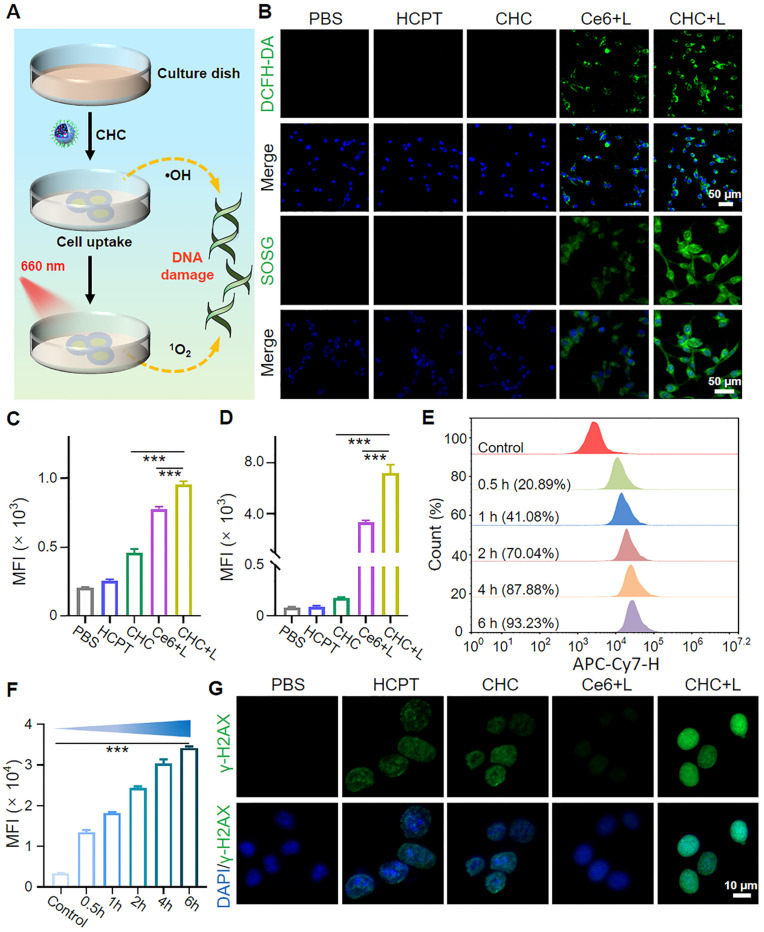
CHC NPs generate radicals and ^1^O_2_ upon 660 nm irradiation to push cell death. (A) Diagram of cellular endocytosis and enhanced DNA damage by producing ROS. (B) Detection of intracellular ROS and ^1^O_2_ generation in CT26 cells through various administration *via* CLSM and mean fluorescence intensity (MFI) of (C) DCFH-DA or (D) SOSG. (E) Flow cytometric profile of CHC NPs for different treatment time and (F) corresponding MFI. (G) The degree of DNA damage in different groups. Data signified as mean ± SD (n = 3).

According to previous researches, irreversible cell damage could emerge under intense oxidative stress because of outrageous high ROS levels, which as a signal to initiate antitumor reaction (Figure 4A). DCFH-DA and SOSG probes were used to respectively detect the intracellular levels of ROS and ^1^O_2_, which could specifically react with them and both produce green fluorescence (Figure 4B). After incubation with CHC NPs for 6 h, NIR 660 nm laser irradiation was operated on CT26 cells for 3 min ahead of visualized by CLSM. Apparently, consistent green fluorescence of CHC+L group was located in the whole cells indicating the elevated ROS and ^1^O_2_ production, which different from other treatment (Figure 4C-D). The above facts implied that CHC NPs could induce the tumor-specific oxidative stress by producing various types of highly toxic reactive oxygen species along with drug toxicity for causing damage to tumor cells.

Owing to the suitable size and neutral potential, the cell uptake modes and intracellular distribution of CHC NPs in CT26 were made a thorough inquiry. CHC NPs chose endocytosis as a viable approach to enter the tumor cells, first nanoparticals attached to cell membrane and membrane invaginated to form vesicles, then CHC NPs-packaged vesicles detached from the membrane to further arrive at lysosomes and other organelles. Concretely, the time-dependent pinocytosis behavior of CHC NPs was recorded by flow cytometry (FCM), approximately 93.23% CHC NPs internalization throughout the whole CT26 cells at 6 h (Figure 4E-F). Colocalization assay and drug distribution were investigated by CLSM with Lyso-Tracker Green or Mito-Tracker Green dye, respectively (Figure S8 and S9). Among the above results indicated that quick uptake efficiency and particularity of CHC NPs made it easy to transport for exerting the combined therapeutic effects of HCPT and Ce6.

To investigate the mechanism of chemotherapeutic drug HCPT with DNA damage response, the DNA damage degree of CHC NPs or free HCPT has been compared in Figure 4G and S10. After incubated with drugs, the intensity of green fluorescence in CHC+L group was strongest in comparison with other treatment, while the group without drug treatment had no fluorescence at all. Since DNA has a certain self-repairing ability during a certain period of time, the strong DNA damage caused by HCPT, cooperated with the violent tumor-specific oxidative stress response induced by CDT and PDT has accelerated cell death.

### CHC NPs induced ICD *in vitro* and *in vivo*

**Figure 5:**
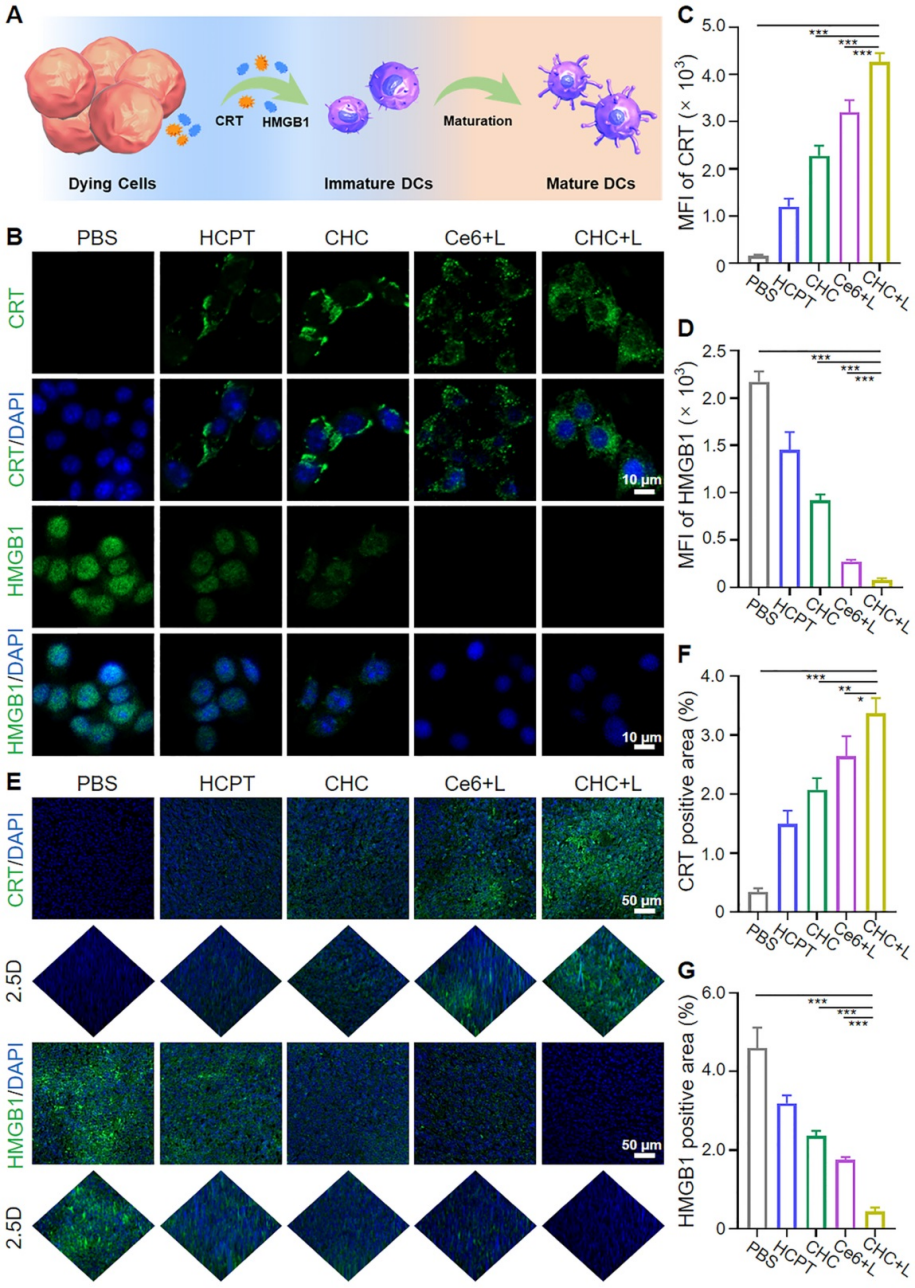
Immunogenic cell death induction of CHC NPs *in vitro* and *vivo*. (A) Schematic illustration of CHC NPs mediated activation of DCs maturation *via* CRT and HMGB1 secretion. (B) CLSM examination of CRT and HMGB1 exposure in CT26 cells with different treatments for 24 h and corresponding fluorescence intensity (C and D). (E) Immunofluorescence staining of CRT and HMGB1 release in CT26 tumor sections and corresponding quantification (F and G). Data displayed as mean ± SD, n = 3.

Photodynamic therapy (PDT) activating immunogenic cell death (ICD) to induce personalized tumor vaccine *in situ* has been verified to expose tumor-associated antigens and recruit immune cells to trigger tumor-specific immune response (Figure 5A). Whether CHC NPs can effectively trigger ICD was investigated *in vitro* by means of the major biochemical indicators, calreticulin (CRT) and high-mobility group box 1 (HMGB1). Transferring from rough endoplasmic reticulum (rER) to cytomembrane surface, CRT is involved in early stage of dendritic cell (DC) maturation induction, and subsequently HMGB1 spreads from the nucleus to the extracellular matrix for participating in the late ICD *via* binding antigen presenting cells (APCs) to speed up DC maturation. Expression of CRT on CT26 cell membrane and extracellular release of HMGB1 *in vitro* were observed and quantified with CLSM (Figure 5B-D). Since the upregulated level of ROS in tumor cells would accelerate CRT translocation and HMGB1 release, CHC NPs displayed superior ROS generation capacity stemming from chemodynamic effect of Fenton reaction and photodynamic effect of Ce6. CHC+L group appeared remarkably stronger CRT green fluorescence and weaker HMGB1 green fluorescence upon 660 nm irradiation. In addition to the evaluation of correlated biomarkers from damaged CT26 cells, tumor tissues under various treatments about CRT expression and HMGB1 migration were sequentially assessed by immunofluorescent staining (Figure 5E). As visualized in Figure 5F and 5G, CHC+L group resulted in the significant exposure of CRT from rER and the insignificant migration of HMGB1 from cell nucleus or cytoplasm to drive autoimmunity. Both *in vitro* and* in vivo* manifestations validated that CHC NPs could serve as optimal ICD-induction agent.

### Biodistribution and penetration capacity of CHC NPs

**Figure 6:**
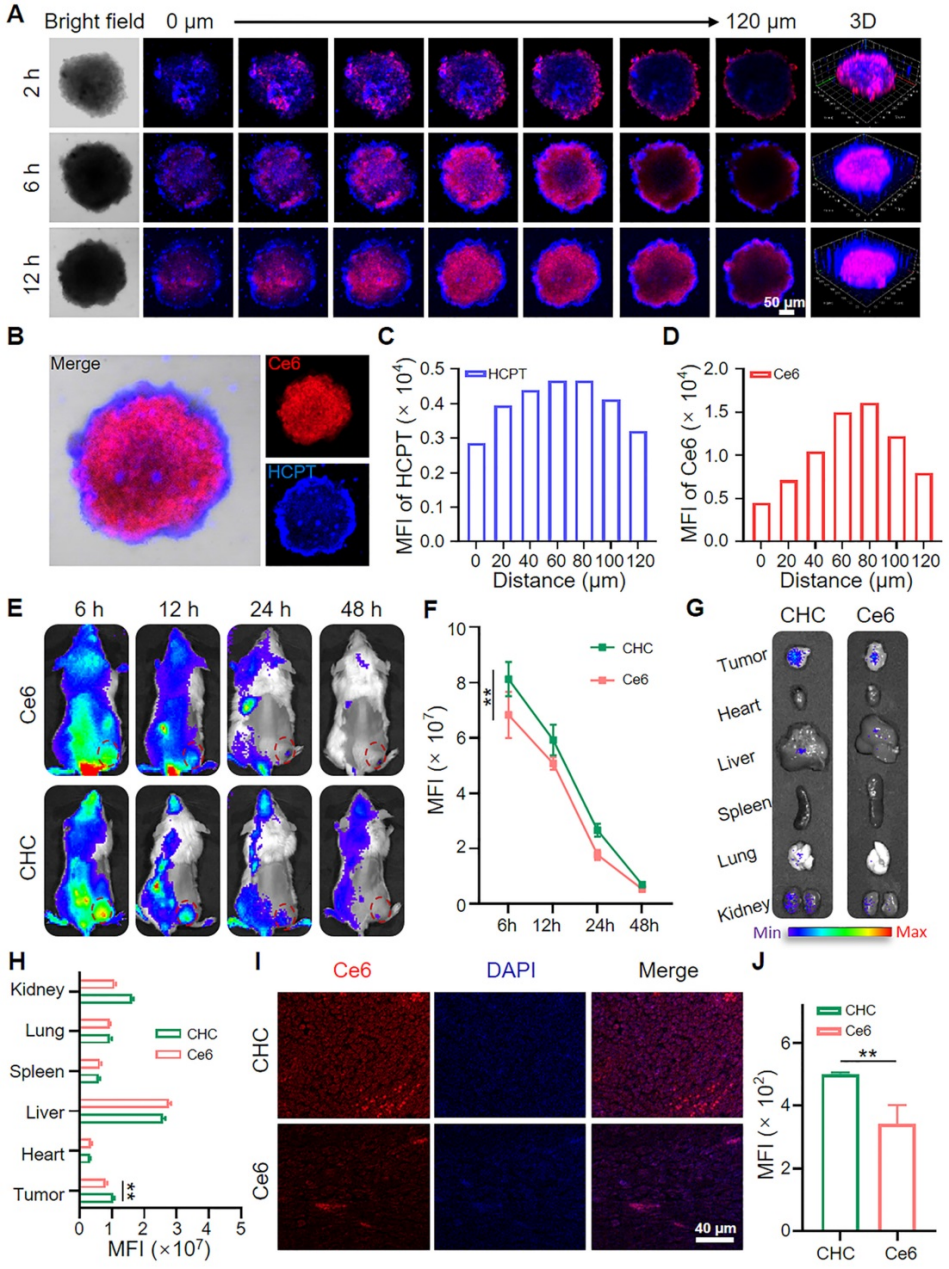
Biodistribution and deep penetration of CHC NPs. (A) Estimate about penetration of CHC NPs *via* CT26 MCSs treated for 2 h, 6 h and 12 h. (B) Individual CLSM images of CT26 MCSs after 12 h treatment by CHC NPs and corresponding fluorescence intensity of (C) HCPT or (D) Ce6. (E) NIR fluorescence photographs of CT26 xenograft mice at various timepoints and (F) statistic comparison of fluorescence intensity in tumors. (G) NIR fluorescence images of major organs at 48 h and (H) quantification of fluorescence intensity in tumor and major organs. (I) Fluorescence imaging of the excised tumors after 48 h post-injection of Ce6 or CHC NPs and (J) mean fluorescence intensity. Data denoted as mean ± SD, n = 3.

After verifying the outstanding efficacy of CHC for ICD effect* in vitro* and *in vivo*, the overall assessment about the performance of drug delivery level from outside to inside was further carried out. Nanodrugs equipped with appropriate size are easier to exhibit EPR effects in comparison with small drug molecules. Using CT26 MCSs model simulated deep penetration of CHC NPs in tumor to estimate the permeability and therapeutic function of nanoparticles (Figure 6A). Chlorin e6 (Ce6), as an auto-fluorescent tracker upon 660 nm irradiation, was involved in fluorescence imaging to determine intracellular localization with the generation of red fluorescence, which agreed with vivid fluorescence emission of Ce6 at 670 nm visualized by fluorescence photometer (Figure 1D), proving the successful loading of Ce6 into CHC NPs. Besides, the blue HCPT fluorescence at different vertical layer was caught by CLSM Z-stack pattern after cultured with CHC NPs. The journey of CHC NPs started from the edge of MCSs at 2 h to nearly filled the entire MCSs at 12 h, was manifested through the change of fluorescence in detail (Figure S11). As described in Figure 6B, CLSM images of CT26 MCSs after 12 h treatment by CHC NPs were displayed in the form of individual channel to clearly distinct and ensure both Ce6 and HCPT encapsulation. The corresponding fluorescence intensity of HCPT and Ce6 at each vertical layer were shown in Figure 6C and 6D. Notably, both the strongest fluorescence intensity of HCPT and Ce6 were viewed at Z-axis distance of 80 µm under 12 h treatment with CHC NPs. Thus, the time-dependent permeation performance also demonstrated that CHC nanoparticles utilized EPR effect to effectively accumulate at tumor sites and gradually permeate.

Meanwhile, biodistribution of CHC NPs *in vivo* was also analyzed on mice bearing CT26 tumor cells, taking advantage of Near-infrared imaging capability of Ce6 to endow fluorescent properties for CHC NPs. As depicted in Figure 6E, the fluorescence intensity of tumor site in CT26-bearing mice reduced with time, but the intensity of CHC always surpassed free Ce6, implying that CHC nanoparticles could be better enriched in tumor site and excreted from the body with the normal metabolism (Figure 6F). In terms of CHC NPs, there was no retention in major organs after 48 h and additional toxic side effects would not be formed (Figure 6G-H). Fluorescence imaging of tumor sections were applied to analyze the permeation and retention of CHC at 48 h, where CHC group still with obvious red fluorescence signal of Ce6 (Figure 6I and 6J). Therefore, CHC NPs exhibited remarkable penetration ability *in vitro* or* in vivo*, which could stay a balance of penetration and retention to elevate the therapeutic efficiency.

### Antitumor effect of CHC NPs *in vivo*

**Figure 7:**
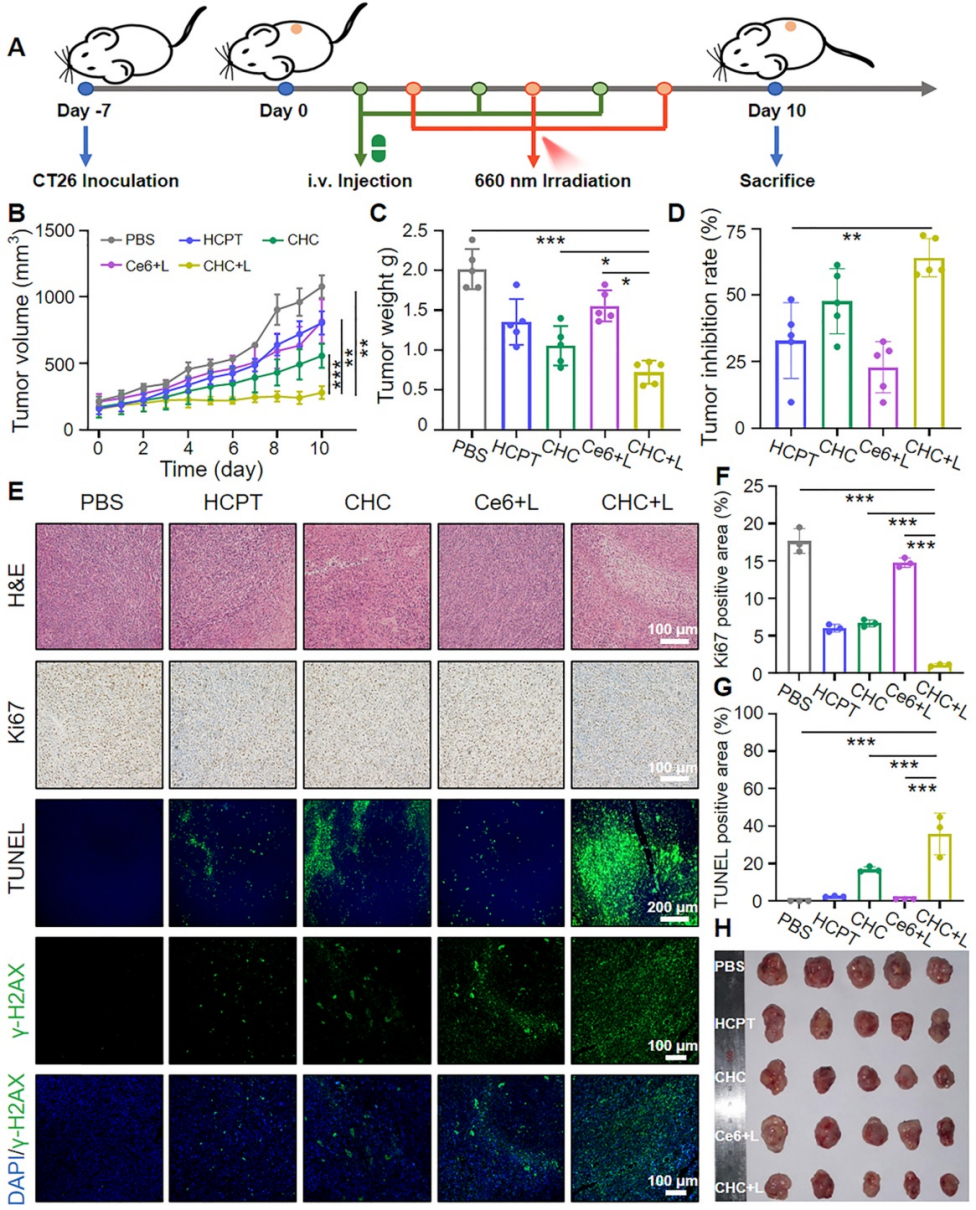
Tumor growth synergistic inhibition of CHC NPs. (A) Sketch map of dual-drug synergy therapy in CT26 xenograft mice. (B) Tumor growth curves and (C) tumor weight at different groups for 10 days. (D) Tumor inhibition rate after 10 days treatment. (E) H&E, Ki67, TUNEL and DNA damage staining of CT26 tumors at the end of treatment. Qualitative analysis of (F) Ki67 generating cells and (G) TUNEL apoptosis cells upon various treatments. (H) Digital photograph of the excised tumors from different groups.

Given aforementioned fantastic treatment outcomes of CHC NPs in CT26 cells by utilizing oxidative lesions and ICD originated from CT/CDT/PDT, murine colon cancer CT26 model was constructed to further seek therapeutic efficacy on Balb/c mice. As present in Figure 7A, CT26 cells were subcutaneously inoculated to models, and CT26-bearing mice were grouped into five treatment types at random, respectively PBS, HCPT, CHC, Ce6+L, CHC+L. The treatment course included intravenous injection of drugs for three times, where groups of Ce6+L and CHC+L exposed to 660 nm laser (180 mW/cm^2^) after 24 h administration. The average tumor volume of mice in PBS group rose perpendicularly from ~218 mm^3^ to ~1078 mm^3^, but CHC+L treatment exhibited exceptional tumor inhibitory effect with significant reduction in tumor mass (Figure 7B). Groups of HCPT and Ce6+L had shown little benefit, however, HCPT and Ce6 loaded CHC NPs treatment was second only to CHC+L. It was mainly because that HCPT could significantly inhibit the genetic material DNA synthesis by suppressing the activity of DNA topoisomerase Ⅰ, but low toxicity compared to CPT. Further calculation of tumor weight and inhibition rate once again illustrated the advantages of mainly chemotherapy combined with CDT/PDT therapies in anti-tumor issues (Figure 7C and D). For tumor tissue sections through H&E staining, CHC+L treated group expressed evident tumor apoptosis/necrosis and the largest damaged area, CHC group appeared a little, while tumor cells of other treatment group were in good condition (Figure 7E). DNA damage was further validated *in vivo* by tumor sections after treatment and mean fluorescence intensity was shown in Figure S12. Ki67 immunohistochemical staining intuitively performed the least brown cell proliferation in CHC+L group, corresponding to the most intensive green fluorescence of apoptotic cells in CHC+L for TUNEL staining. The positive area quantification of Ki67 and TUNEL were displayed in Figure 7F and 7G in turns. During 10 days treatment, the body weight among the all groups had reasonable fluctuation and the digital photograph of the excised tumors from various administration groups as expected also witnessed the remarkable efficacy of CHC+L (Figure 7H and S13). Aforesaid results all confirmed the serious apoptosis induced by CHC+L group to emphasize the prominent therapy efficiency of CHC NPs. Beyond that, H&E sections of major organs in CHC+L group had no tumor metastasis by contrast with other treatment (Figure S14).

### Biosafety assessment

The primary prerequisite for CHC NPs to be transformed into clinical application in the future is excellent biological safety. Hemocompatibility is regarded as an important target to evaluate the effect of pharmaceutical preparations on blood stability by CHC NPs and erythrocyte cocultivation. We selected the concentration range of Ce6 in CHC from 0 to 200 μg/mL for hemolysis experiment, and all calculation results were under 2% (Figure S15A). The ultraviolet absorption peaks of hemoglobin appearing at 541 nm and 574 nm were measured by UV-Vis, where red blood corpuscles were severely damaged by Triton X-100 as the positive control group with exceptionally high absorbance, illustrating the prominent hemocompatibility and performance stability of CHC NPs for ensuring the normal blood circulation (Figure S15B). As for the biocompatibility of CHC NPs and murine L929 fibroblasts (L929 cells), MTT assay results showed the cell survival rate of L929 was above 89% at various concentration, indicating CHC as a superior biocompatible nanodrug (Figure S16). Furthermore, the biosafety of CHC NPs conduct *in vivo* was viewed as one of the vital contents. Undergoing intravenous injection of different reagents (PBS, HCPT, Ce6 and CHC NPs, respectively), the body weight of mice fluctuated within the normal range (Figure S17). The parameters of blood routine incorporating WBC, LYM, RBC, HGB, HCT, MCHC, RDW, PLT and MPV were investigated and no significant toxicity or inflammatory response in various treatment groups after 1 day, 7 days and 14 days (Figure S18). These analysis reports manifested that CHC NPs could suppress tumor growth *via* synergistic chemo-chemodynamic/photodynamic therapy with outstanding biosafety application.

## Conclusion

This study launched an efficient and environmental-friendly process that calcium carbonate hybrid nanoparticles equipped with classic chemotherapy drug HCPT and photosensitizer Ce6 were successfully prepared, achieving chemotherapy-based, synergetic CDT/PDT therapy to trigger immunogenic death for eradicating tumors. The introduction of PEG-P(Glu) block copolymers greatly improved and optimized hydrophilicity and stabilization of CHC NPs, to facilitate the transfer of nanoparticles *via* the blood circulation and enrich at tumor sites through EPR effect. In addition, CHC NPs were manifested to trigger high ROS yield under CDT/PDT activation due to the internal stimulation of Fenton reaction to generate •OH and the production of ^1^O_2_ upon utilizing exogenous stimulation of 660 nm laser. Meanwhile, ICD was efficiently induced to promote antitumor immunogenicity for eliminating malignancy. Exploiting structure of calcium carbonate decomposed autonomously at low pH in tumor microenvironment, the proposed composite mode of chemotherapy and CDT/PDT for cancer therapy was found at the first time to inspire more related designs on the strength of responsive combination therapy, in order that promote the transformation of nanomedicine into clinic.

## Supplementary Material

Supplementary figures.
