# Supplementary material for: Polyamino acid calcified nanohybrids induce immunogenic cell death for augmented chemotherapy and chemo-photodynamic synergistic therapy
Source: Theranostics. 2021 Sep 21;11(19):9652–66. doi: 10.7150/thno.64354 (PMC8490510; doi:10.7150/thno.64354)
Supplement: Supplementary file 1 — Supplementary figures. [file thnov11p9652s1.pdf]

Supporting Information

Polyamino acid calcified nanohybrids induce immunogenic cell death for augmented chemotherapy and chemo-photodynamic synergistic therapy

Wei Qiu[a], Mengyun Liang[a], Yuan Gao[a], Xuelian Yang[a], Xingyao Zhang[a], Xiaoli Zhang[b]*, Peng Xue[a], Yuejun Kang[a] and Zhigang Xu[a,c]*

[a]Key Laboratory of Luminescence Analysis and Molecular Sensing (Southwest University), Ministry of Education, School of Materials and Energy and Chongqing Engineering Research Center for Micro-Nano Biomedical Materials and Devices, Southwest University, Chongqing 400715, P. R. China

[b]Pediatric Research Institute, Department of Hematology and Oncology, Shenzhen Children's Hospital, Shenzhen, Guangdong 518038, P. R. China

[c]Key Laboratory of Laser Technology and Optoelectronic Functional Materials of Hainan Province, College of Chemistry and Chemical Engineering, Hainan Normal University, Haikou 571158, P. R. China

*Corresponding authors: zgxu@swu.edu.cn (Z. Xu); xlzhang82@hotmail.com (X. Zhang)

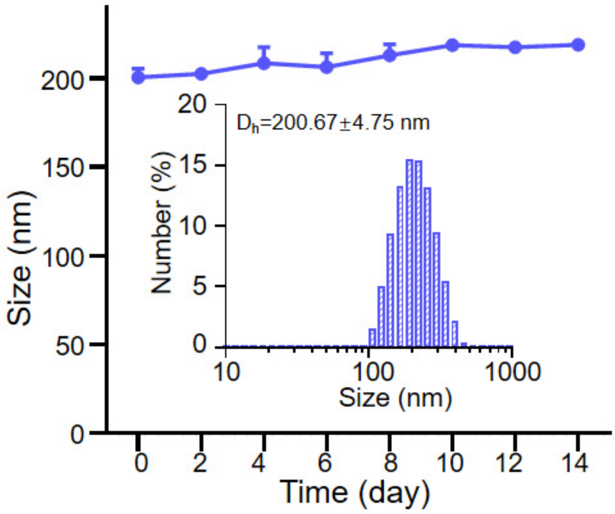

Figure S1. Hydrodynamic diameter of CHC NPs in PBS with 10% FBS during 2 weeks, as measured by DLS.

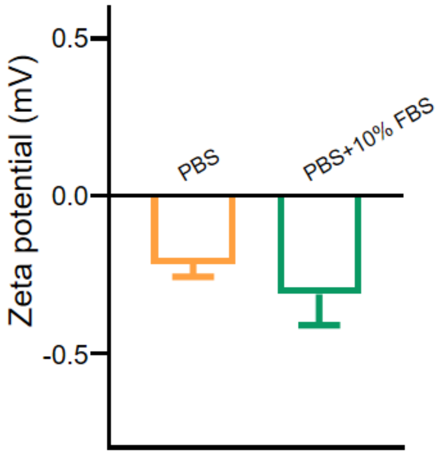

Figure S2. Zeta potentials of CHC NPs in PBS with or without 10% FBS.

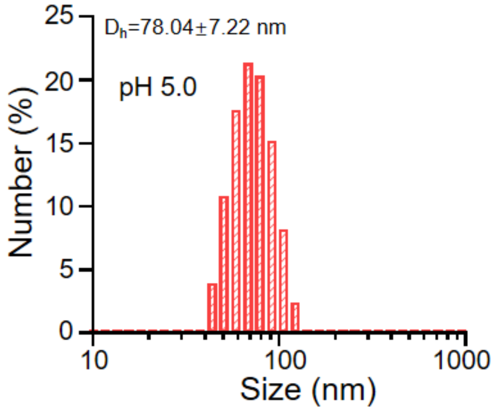

Figure S3. Size distribution of CHC NPs in acid PBS solution with pH 5.0 after 24 h treatment time.

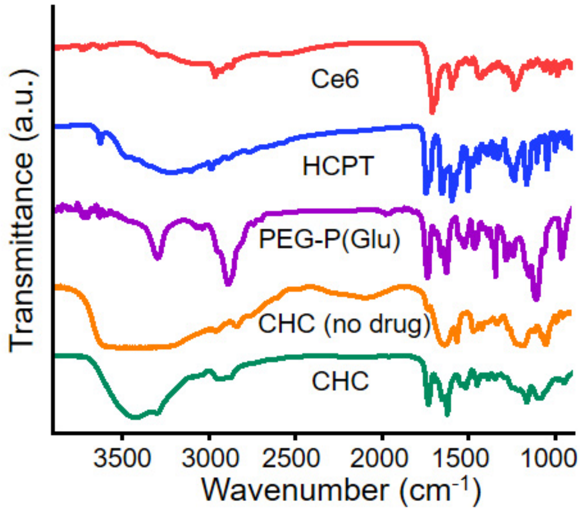

Figure S4. FT-IR spectra of every component from CHC NPs.

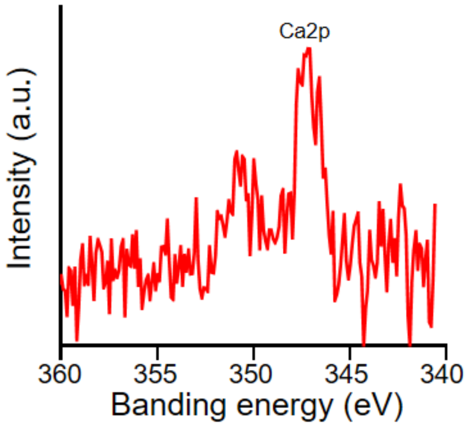

Figure S5. Core level XPS spectrum of Ca2p in CHC NPs.

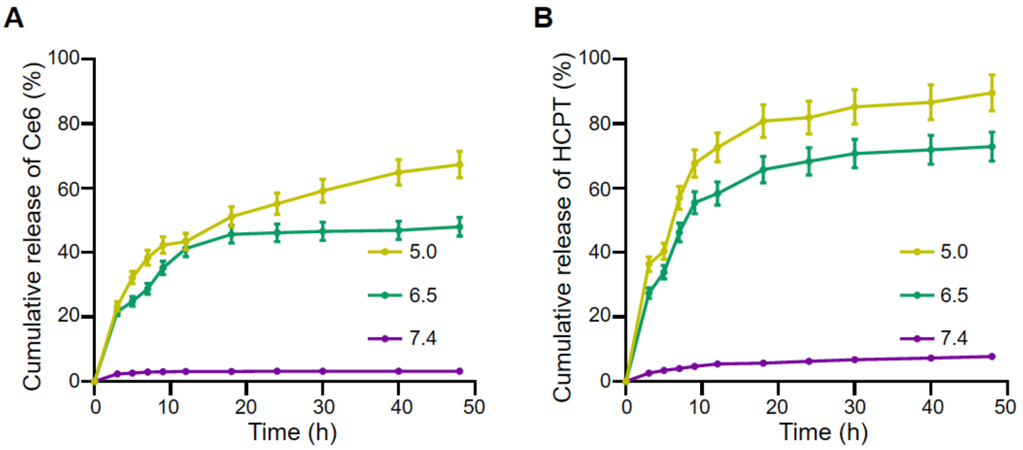

Figure S6. In vitro release pattern of (A) Ce6 and (B) HCPT from CHC NPs in different pH condition.

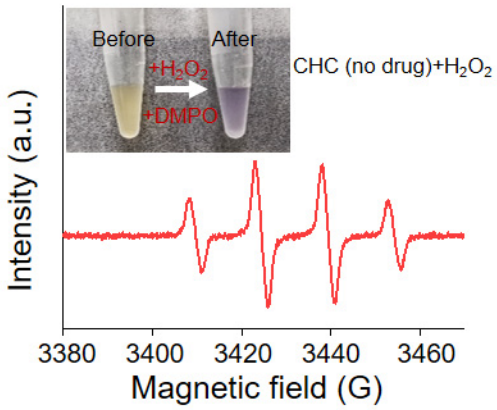

Figure S7. ESR spectrum of DMPO/•OH in CHC NPs without HCPT or Ce6.

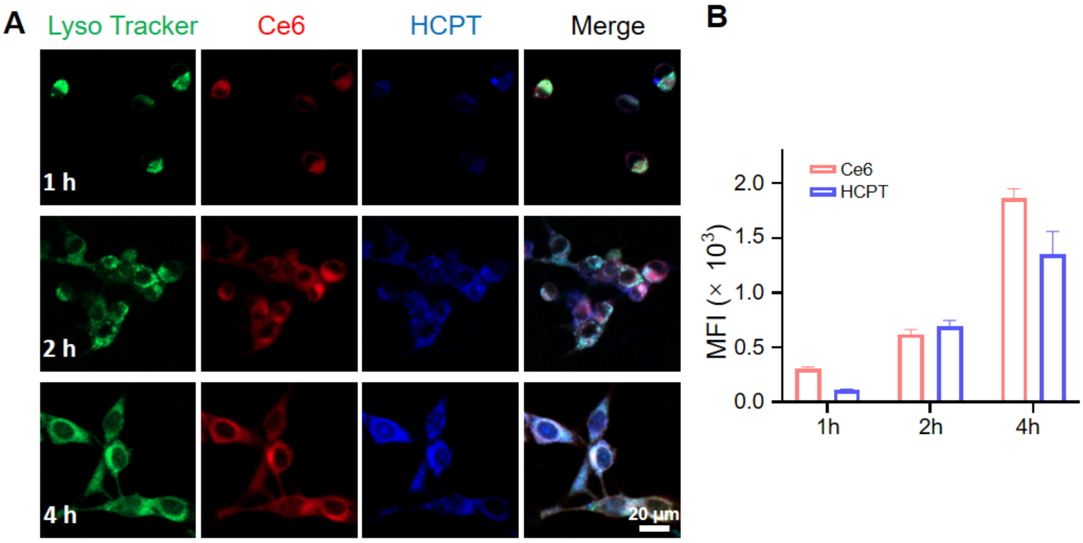

Figure S8. (A) CLSM images and (B) quantitative results of lysosomal colocalization in CT26 cells. Lysosome was stained with Lysotracker Green (green); red (Ce6) and blue (HCPT) fluorescence derived from CHC NPs.

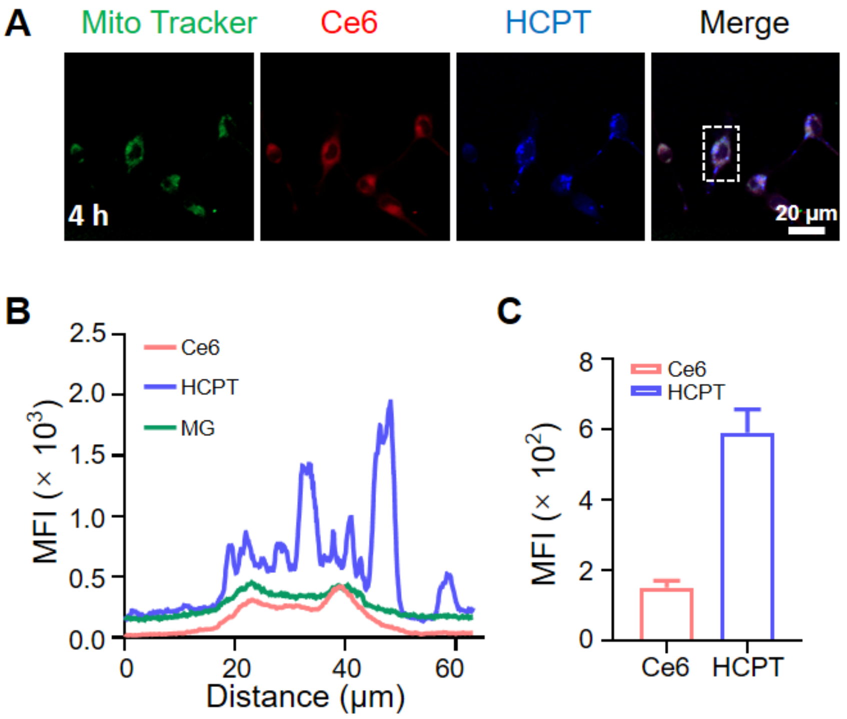

Figure S9. (A) Mitochondria colocalization images of CT26 cells stained with Mito-tracker Green (MG). (B) Dynamic fluorescence intensity and (C) fluorescence intensity of Ce6 and HCPT at 4h.

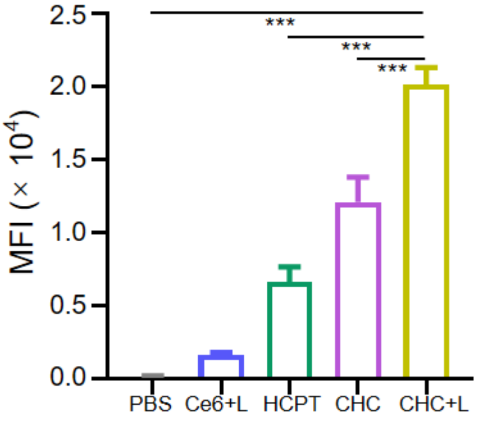

Figure S10. Quantification of DNA damage at cell level.

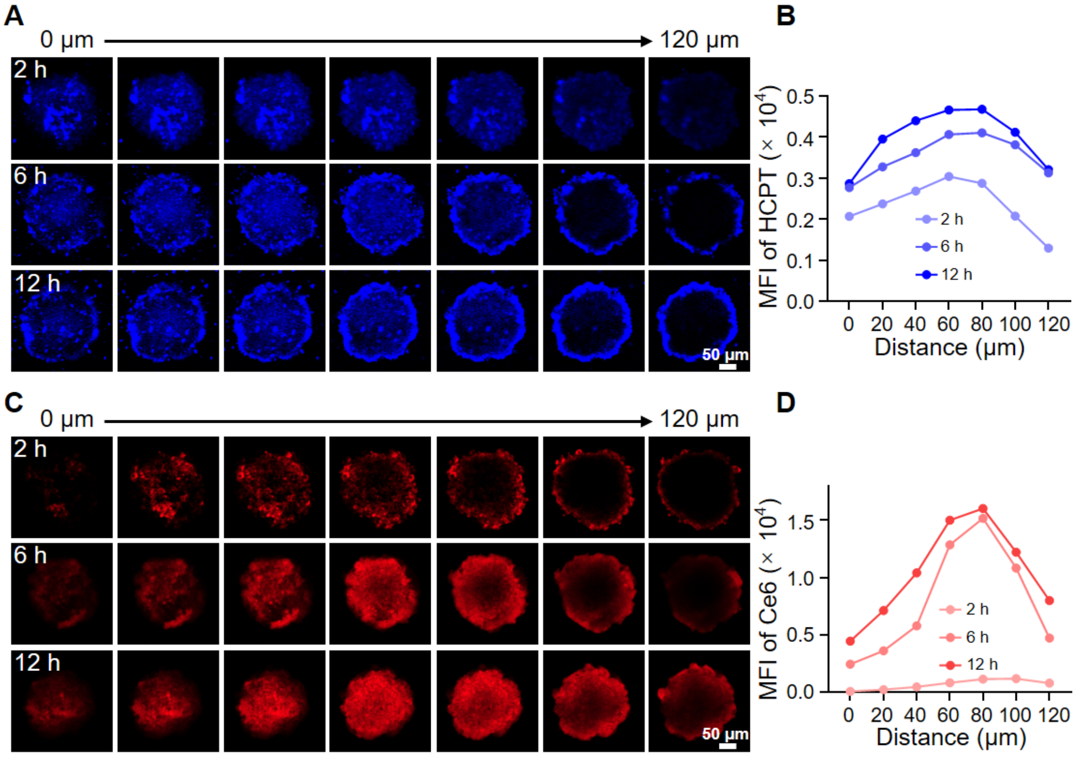

Figure S11. Penetration of HCPT and Ce6 from CHC NPs in CT26 MCSs for 2 h, 6 h and 12 h, respectively. (A) Images of HCPT at each layer. (B) Mean fluorescence intensity of HCPT with different depth. (C) Images of Ce6 at each layer. (D) Mean fluorescence intensity of Ce6 with different depth.

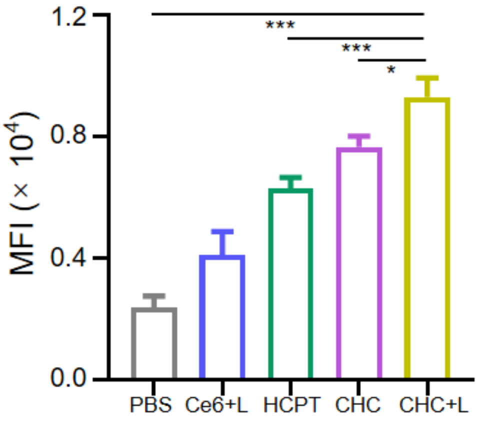

Figure S12. Mean fluorescence intensity of DNA damage in tumor sections.

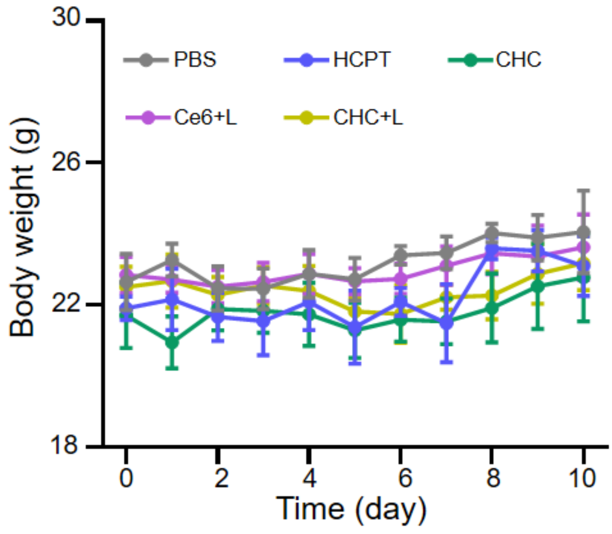

Figure S13. Body weight at different groups for 10 days synergistic therapy.

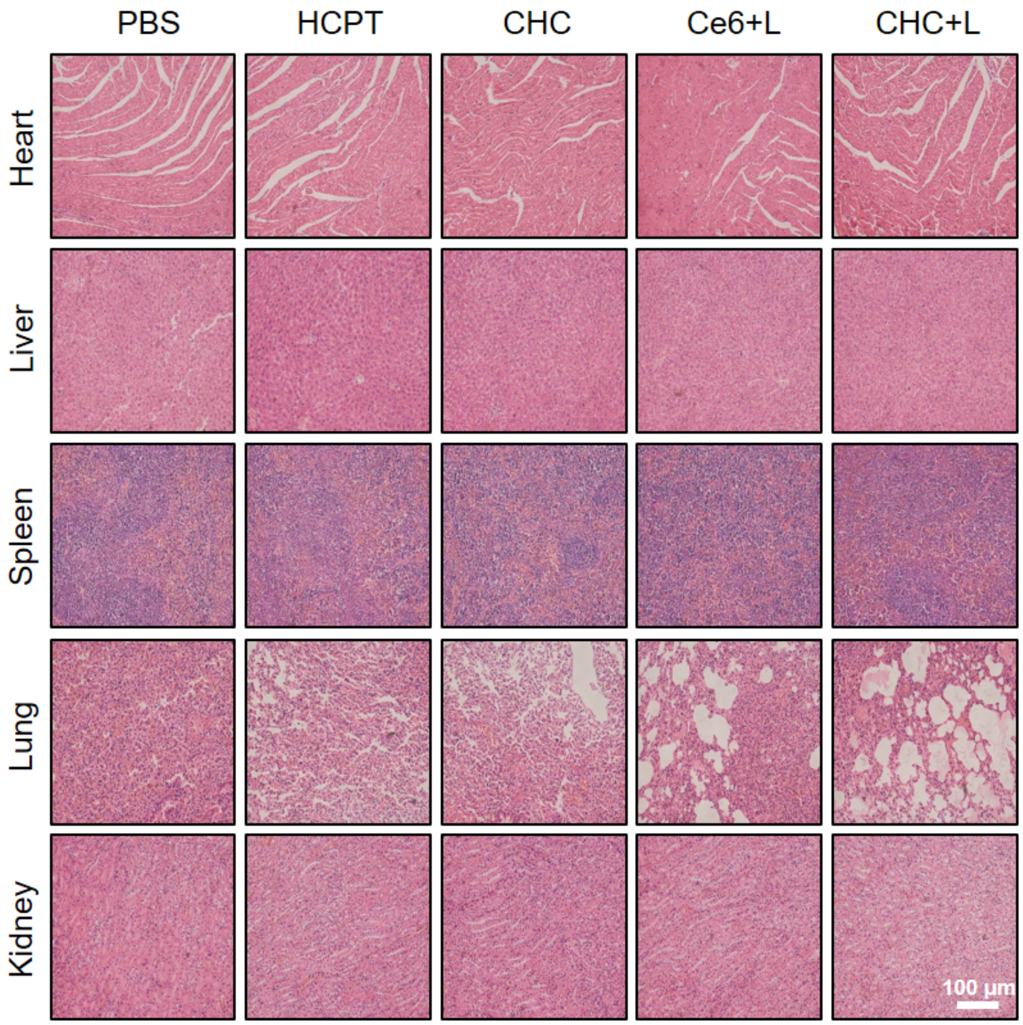

Figure S14. H&E staining about major organs of CT26 mice after various treatments.

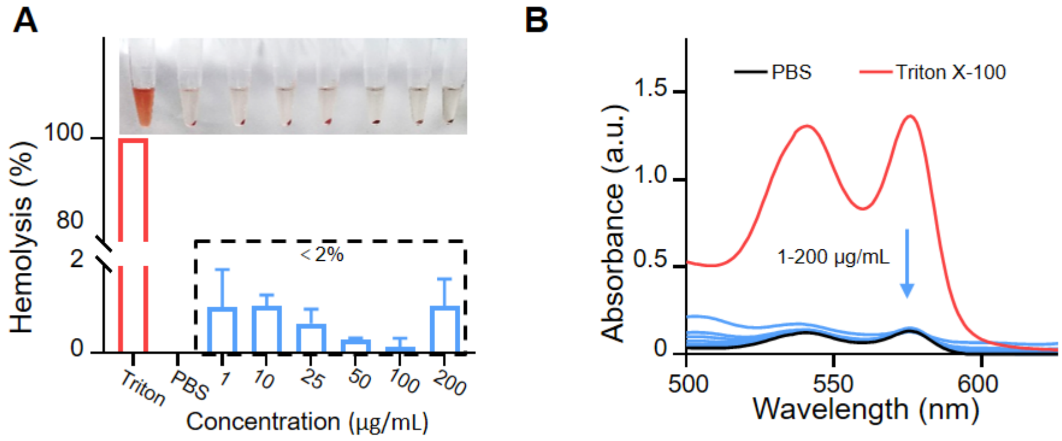

Figure S15. (A) The hemolysis rate of CHC NPs at different concentration in red blood cells and (B) UV-vis absorption of hemoglobin.

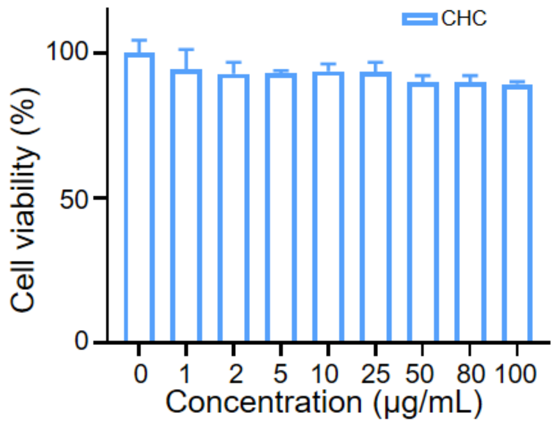

Figure S16. Biosafety of CHC NPs by MTT assay with L929 cells.

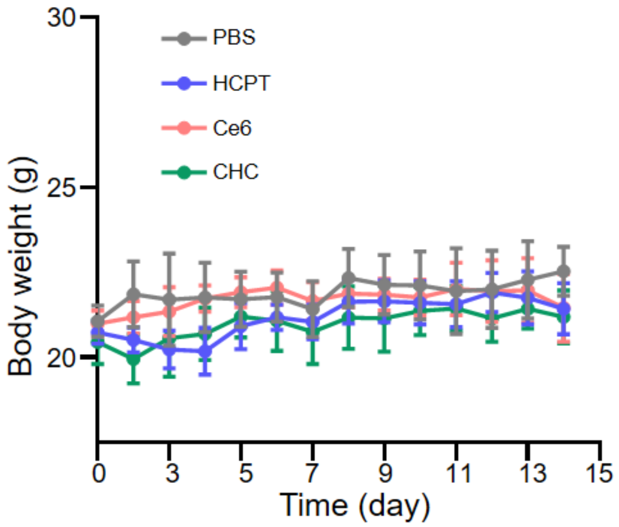

Figure S17. Body weight of mice during routine blood test.

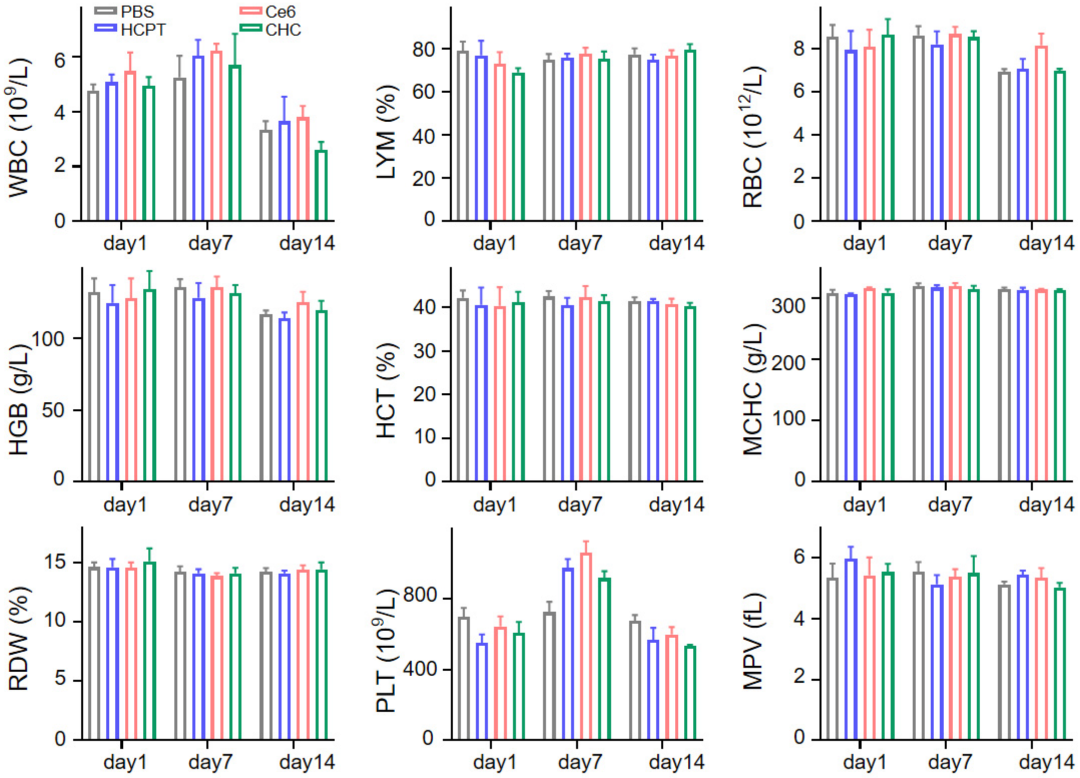

Figure S18. Analysis of peripheral blood from mice injected with different drugs for 1 day, 7 days or 14 days. Blood detection parameters: WBC, LYM, RBC, HGB, HCT, MCHC, RDW, PLT and MPV. Data signified as mean ± SD (n = 3).
